# Out-of-hospital therapeutic hypothermia in cardiac arrest victims

**DOI:** 10.1186/1757-7241-17-52

**Published:** 2009-10-12

**Authors:** Wilhelm Behringer, Jasmin Arrich, Michael Holzer, Fritz Sterz

**Affiliations:** 1 Department of Emergency Medicine, Medical University of Vienna, Vienna General Hospital, Waehringer Guertel 18-20, 1090 Vienna, Austria

## Abstract

Despite many years of research, outcome after cardiac arrest is dismal. Since 2005, the European Resuscitation Council recommends in its guidelines the use of mild therapeutic hypothermia (32-34°) for 12 to 24 hours in patients successfully resuscitated from cardiac arrest. The benefit of resuscitative mild hypothermia (induced after resuscitation) is well established, while the benefit of preservative mild to moderate hypothermia (induced during cardiac arrest) needs further investigation before recommending it for clinical routine. Animal data and limited human data suggest that early and fast cooling might be essential for the beneficial effect of resuscitative mild hypothermia. Out-of-hospital cooling has been shown to be feasible and safe by means of intravenous infusion with cold fluids or non-invasively with cooling pads. A combination of these cooling methods might further improve cooling efficacy. If out-of-hospital cooling will further improve functional outcome as compared with in-hospital cooling needs to be determined in a prospective, randomised, sufficiently powered clinical trial.

## Background

Sudden cardiac arrest remains a major unresolved public health problem. In Europe and the USA, approximately 425.000 people suffer of sudden cardiac death with very poor survival, usually less than 10% [1,2]. After cardiac arrest and brain ischemia, reperfusion initiates multiple independent chemical cascades and fatal pathways, resulting in neuronal death due to necrosis and apoptosis [3]. Because of the multi-factorial pathogenesis of post-arrest neuronal death, a multifaceted treatment strategy is required to achieve survival without brain damage. Hypothermia, a re-discovered promising treatment strategy, exerts its beneficial effects on brain ischemia by various mechanisms, and perfectly fulfils the requirements of a multifaceted treatment strategy [4].

In therapeutic hypothermia, different degrees of cooling can be differentiated, though definition of these temperature levels may differ slightly between authors: mild (34 to 32°C), moderate (31 to 28°C), deep (27 to 11°C), profound (10 to 6°C), and ultra-profound (5 to 0°C) hypothermia. Protective hypothermia, induced before cardiac arrest, has to be differentiated from preservative hypothermia, induced during cardiac arrest treatment, and from resuscitative hypothermia, induced after successful resuscitation. Protective hypothermia is used in cardiac surgery and neurosurgery, but is clinically unrealistic in sudden cardiac death. This review will focus on a) preservative mild hypothermia during cardiac arrest treatment and b) resuscitative mild hypothermia after successful resuscitation in respect to its clinical application in the out-of-hospital setting.

## Preservative hypothermia

Preservative hypothermia can further be differentiated into the induction of hypothermia during ischemia (before initiation of resuscitation - or before reperfusion) and the induction of hypothermia during resuscitation.

### Induction of hypothermia during ischemia, before resuscitation

Research in myocytes showed that injury to cells not only occurs during ischemia itself, but mainly with reperfusion by initiating several cascades leading to cell death [5-7]. Besides other effects, intra-ischemic hypothermia attenuates the inflammatory response [8], oxidative DNA damage and DNA damage-triggered pro-death signalling after resuscitation [9]. In various animal studies using vessel occlusion or cardiac arrest models, the induction of hypothermia already during cardiac arrest (before the start of resuscitation) improved outcome as compared with hypothermia induced after successful resuscitation [10-17]. Importantly, a delay of resuscitation efforts to allow establishment of hypothermia before reperfusion, did not affect the beneficial effect of hypothermia on cardiac function and survival [18,19].

The induction of hypothermia during ischemia, before resuscitation, is an intriguing concept, but reserved for experimental animal studies. Before bringing this concept into clinical reality, many questions need to be answered: how long can resuscitation be delayed for the purpose of inducing hypothermia? Which level of hypothermia has to be induced? How long to maintain a certain level of hypothermia before re-warming?

### Induction of hypothermia during resuscitation

Induction of hypothermia during resuscitation is a more realistic clinical scenario, because resuscitation does not have to be delayed for induction of hypothermia. In a swine cardiac arrest model, induction of mild hypothermia with beginning of resuscitation improved resuscitabilty, but not short term neurologic outcome [20]; mild hypothermia was induced with an i.v. infusion of 30 ml/kg 4° cold saline. In another swine study, surface cooling to 34°C during the first 30 minutes of prolonged resuscitation increased rate of restoration of spontaneous circulation [21]. In a dog cardiac arrest model, induction of mild hypothermia with veno-venous blood shunt cooling during prolonged cardiac arrest improved neurologic outcome as compared to normothermia [22], but hypothermia had to be induced very early during resuscitation, otherwise its beneficial effect was diminished [23].

Only three explorative human studies investigated the feasibility of cooling during resuscitation in the out-of-hospital setting [24-26]. In the study by Bruel et al [24], hypothermia was induced in 33 patients by i.v. infusion of 2 l of 4°C normal saline 0.9% over 30 minutes with pressure bags during advanced life support prior to arrival at the hospital; the oesophageal temperature decreased significantly by -2.1°C ± 0.29°C after cooling to a median temperature of 33.3°C (IQR 32.3-34.3); twenty (61%) of the patients were successfully resuscitated, in whom mild hypothermia (<34°C) was achieved 16 min (IQR 12-25) after ROSC; the time delay to start cooling, and how many patients have achieved ROSC before the total volume was infused, were not reported; one patient developed pulmonary oedema; 3 (9%) patients survived with good neurologic outcome. The other two studies were performed by Kämäräinen et al [25,26]. Since the second study [26] includes patients of the first study [25], only the second study will be discussed here: hypothermia was induced in 17 patients by i.v. infusion of 4°C Ringers acetate with a rate of 50 ml/min during resuscitation, and a rate of 100 ml/min after resuscitation until a nasopharyngeal temperature of 33°C was achieved. Cooling was started at 12 min after start of CPR at initial nasopharyngeal temperature of 35.17 ± 0.57°C. Temperature on hospital admission was 33.83 ± 0.77°C (-1.34°C, p < 0.001). Mean infused volume of cold fluid was 1571 ± 517 ml. Thirteen (76%) of the patients were successfully resuscitated, and 1 (6%) patient survived with good neurologic outcome.

These preliminary studies [24-26] proved the feasibility of inducing mild hypothermia during resuscitation with i.v. infusion of 4° cold fluids in cardiac arrest patients. But there is no human outcome data today to support the use of volume loading during resuscitation in daily clinical practice. The influence of volume load during resuscitation on resuscitabilty has to be evaluated first in large animal outcome studies. It remains to be determined whether the potential beneficial effect of hypothermia on neurologic function is offset by a deleterious effect on survival. Temperature regulation with infusion of cold fluids during low-flow has to be considered: in the first study by Kämäräinen et al [25], the lowest mean temperature was 31.7°C, which is lower as the recommended target range of 32-34°C. Volume load during resuscitation might increase right arterial pressure, which might result in reduced vital organ perfusion [27], and thereby worse outcome.

## Resuscitative hypothermia

Already in the 1960s, Peter Safar recommended the use of resuscitative mild hypothermia after successful resuscitation from cardiac arrest in his ABC of post cardiac arrest care [28]. Resuscitative hypothermia research was then given up for 25 years, because experimental and clinical trials had been complicated by the injurious systemic effects of total body cooling. In 2002, results of two prospective randomised clinical trials showed that mild hypothermia initiated after resuscitation from ventricular fibrillation improved survival and neurologic outcome in cardiac arrest survivors compared to patients treated with normothermia [29,30]. In 2005, the guidelines of the European Resuscitation Council recommended: *"Unconscious adult patients with spontaneous circulation after out-of-hospital ventricular fibrillation cardiac arrest should be cooled to 32-34°C. Cooling should be started as soon as possible and continued for at least 12-24 h. Induced hypothermia might also benefit unconscious adult patients with spontaneous circulation after out-of-hospital cardiac arrest from a non-shockable rhythm, or cardiac arrest in hospital" *[31].

The deleterious cascades of neuronal death start already during cardiac arrest, but are boosted with start of reperfusion [3]. In view of the pathophysiology on how neurons die, it would be logical to start with mild hypothermia treatment as soon as possible after resuscitation. In fact, animal studies show consistently that a delay in cooling negates the beneficial effect of mild hypothermia after cardiac arrest [32-35]. Based on these animal studies and on the pathophysiologic mechanisms of cell death, the 2005 guidelines of the European Resuscitation Council recommended: "... *Cooling should be started as soon as possible *..." [31]. Concerning human data, the evidence for the importance of timing of cooling is very limited. One retrospective human study in 49 patients showed in multivariate analyses that any hour delay till coldest temperature or target temperature tended to worsen the likelihood for a favourable outcome by approximately 27% or 31%, respectively [36]; this study did not prove the importance of early cooling, but rather indicates the importance of fast cooling once initiated: time to start cooling was delayed, and did not differ between the patients with good outcome and poor outcome (both groups median of 150 minutes), but there was a statistically significant difference in time to coldest temperature of median 443 minutes in patients with good outcome as compared with median 555 minutes in patients with poor outcome. However, in an observational study in 975 patients after cardiac arrest, time to initiation of therapeutic hypothermia and time to reach the goal temperature had no significant association with outcome [37].

If the recommendations of the European Resuscitation Council [31] were followed, treatment with mild hypothermia might have to be started already in the out-of-hospital setting. The cooling methods for induction of mild hypothermia in the out-of-hospital setting need to be easy to use in order not to distract paramedics and physicians from other aspects of post-resuscitation care (timely transport to the hospital, ventilation, blood pressure control, and others); and at the same time, cooling methods should effectively decrease temperature. For in-hospital cooling, rapid intravenous infusion of cold fluids after cardiac arrest is well tolerated and feasible for induction of mild hypothermia [38-42]. In addition to infusion of cold fluids, various non-invasive [29,43-47] and invasive cooling methods are available [43,44,48-50], but these cooling devices are heavy, bulky, and need energy supply during use, which makes them unsuitable for use in the out-of-hospital setting.

Easy to use non-invasive cooling methods for induction of mild hypothermia in the out-of-hospital setting include simple ice-packs [30,51,52] or cooling pads, which adhere to the patients skin [53]. Ice bags have only limited cooling capacity [51], are cumbersome to use [54], and might result in unintentional overcooling [55]. Recently, a new cooling pad was introduced, which is stored at -2°C in a mobile cooling box in the ambulance car [53]: in this study, cooling was initiated at a median 12 minutes (IQR 8.5-15) after restoration of spontaneous circulation, and an oesophageal temperature of 33°C was achieved within median 70 minutes (IQR 55-106) after start of cooling, with a median cooling rate of 3.3°C/hour (IQR 2.0-4.0).

Infusion of cold fluid after successful resuscitation is also an easy to use cooling method in the out-of-hospital setting [56,57]. In the first study investigating feasibility of cold infusion during transport to the hospital [56], 13 patients were treated with 30 ml/kg of ice-cold Ringer's acetate intravenously with an infusion rate of 100 ml/min; oesophageal temperature decreased by 1.8°C, from 35.8°C to 34.0°C at admission; no results were given on the temperature course after admission. In another study [57], a total of 125 patients were randomized to receive standard care with or without intravenous field cooling; of the 63 patients randomized to cooling, 49 (78%) received an infusion of 500 to 2000 mL of 4°C normal saline before hospital arrival; these 63 patients experienced a mean temperature decrease of 1.2 ± 1.0°C with a hospital arrival temperature of 34.7°C, whereas the 62 patients not randomized to cooling experienced a mean temperature increase of 0.1 ± 0.9°C (P < 0.0001) with a hospital arrival temperature of 35.7°C; magnitude of temperature decrease correlated with the amount of volume infused; no adverse consequences in terms of blood pressure, heart rate, arterial oxygenation, evidence for pulmonary oedema on initial chest x-ray, or re-arrest were reported; moreover, the volume load might aid to hemodynamic improvement. The authors of this study [57] reported also on outcome, there was a trend for awakening and discharged alive from hospital only in ventricular fibrillation patients; main limitations of this study were that not all patients received the full amount of cold fluid, and that patients may or may not have been treated with mild hypothermia in the receiving hospital. These limitations prevent to draw any conclusion of the potential beneficial effect of early cooling on neurologic outcome.

Using cold intravenous fluids might have some limitations: to be effective, infusion must be fast [41], requiring large-bore cannula access which might not be available in all cardiac arrest cases; pulmonary oedema would contraindicate the application of fluids; and re-warming is rapid within 60-90 minutes, requiring an additional cooling method to maintain mild hypothermia [40,41,58]. Combination of cold intravenous fluids with cool-packs or cooling pads in the out-of-hospital setting might overcome these limitations and should be evaluated in further clinical trials.

The potential benefits of starting the cooling process already in the out-of-hospital setting are not limited to duration of cooling during driving time from scene to hospital. In Vienna, average driving time from scene to the emergency department is only 10 minutes (personal communication with the Medical Director of the Vienna Ambulance System); but in the Vienna study with out-of-hospital cooling [53], time from successful resuscitation to arrival at the emergency department was 45 minutes. This includes stabilisation of patient, and transport of patient from the actual location of cardiac arrest (could be the 5^th ^floor of an apartment house without elevator) to the ambulance car. At arrival at the emergency department, cooling had been in progress for 30 minutes and had decreased oesophageal temperature to 35.4°C; target temperature of 33°C was reached within 91 minutes. In the hospital, time to start cooling takes considerably longer: in an Europe-wide multicentre registry of 465 patients treated with hypothermia after cardiac arrest, cooling was initiated at 131 minutes after resuscitation, with a cooling rate of 1.1°C/h [43]. In two other in-hospital studies, time to start cooling was 120 minutes [46], and 95 minutes respectively [48]. Thus, with out-of-hospital cooling, delay of in-hospital cooling was prevented, and target temperature of 33°C was not only reached considerably faster as compared to in-hospital cooling, target temperature was reached, even before cooling could be initiated in the emergency department [53]. If early cooling in the out-of-hospital setting will improve neurologic outcome needs to be investigated in a prospective, randomized, and sufficiently powered clinical trial.

## Conclusion

Since 2005, the European Resuscitation Council recommends in its guidelines the use of mild therapeutic hypothermia (32-34°) for 12 to 24 hours in patients successfully resuscitated from cardiac arrest. The benefit of resuscitative hypothermia (induced after resuscitation) is well established, while the benefit of preservative hypothermia (induced during cardiac arrest) needs further investigation before recommending it for clinical routine. Animal data and limited human data suggest that early and fast cooling might augment the beneficial effect of resuscitative mild hypothermia. Out-of-hospital cooling was shown to be feasible and safe by means of infusion with cold saline or non-invasively with cooling pads. A combination of these cooling methods might further improve cooling efficacy. If out-of-hospital cooling will improve functional outcome as compared with in-hospital cooling needs to be determined.

Despite all the knowledge about hypothermia acquired up to day, additional studies are needed to better define the optimal depth and duration of hypothermia, the role of sedatives and paralytics during cooling, and the optimal re-warming rate after cooling, and to improve the techniques for inducing hypothermia. We strongly encourage joining the international hypothermia network  to enable properly powered, prospective, randomized trials to address all these issues.

## Competing interests

WB is co-founder, share holder, and paid medical consultant of the company EMCOOLS (Vienna, Austria); he holds part of the patent on EMCOOLSpad (EMCOOLS, Vienna, Austria).

JA was employed by grant money from ALSIUS (Irvine, CA, USA), and received speakers honoraria from EMCOOLS (Vienna, Austria).

MH received grant money from Life Recovery Systems (Kinnelon, USA), Kinetic Concepts International (Amstelveen, Nederlands) and Alsius Corp. (Irvine, USA) and speakers honoraria from Kinetic Concepts International (Amstelveen, Nederlands) and Medivance (Louisville, USA).

FS holds part of the patent on EMCOOLSpad (EMCOOLS, Vienna, Austria).

## Authors' contributions

All authors have equally been involved in drafting the manuscript and revising it critically for important intellectual content; and have given final approval of the version to be published.
